# Maim, death cases following the installation of vascular access devices filed with specialized forensic medicine commissions of Tehran, Iran, 2013-2018, (A descriptive study)

**DOI:** 10.22088/cjim.13.4.689

**Published:** 2022

**Authors:** Fares Najari, Jalaluddin Khoshnevis, Zahra Javaheri, Dorsa Najari, Sahar Mirzaei

**Affiliations:** 1 Clinical Research Development Center of Shohada-ye-Tajrish Hospital, Vice- Chancellor in Research Affairs, Shahid Beheshti University of Medical Sciences, Tehran, Iran

**Keywords:** Malpractice, Central venous catheters, Legal medicine, Judicial

## Abstract

**Background:**

Implantation of vascular access devices is of great importance in critically ill patients or those vulnerable to clinical worsening. The aim of this study was to identify the complications of implantation of vascular access leading to complaints from patients, in the forensic medicine commissions of Tehran.

**Methods:**

The present descriptive-cross sectional study was performed on all cases that died from implantation of vascular access devices and complaints about permanent local complications caused by this procedure, filed with the forensic medicine commissions of Tehran in period of 2013-2018, based on selected variables, and the results were statistically analyzed using the chi-square and Fisher exact tests in SPSS Version 18. A p-value of <0.01 was considered statistically significant.

**Results:**

All alleged deaths were due to implantation of central venous port placed in a large vein in the neck, and most organ failure cases were attributed to anterior forearm deformity. The most common cause of death was acute cardiac death; internal bleeding was observed in 14% of them. Most complaints of death were filed against general surgery and anesthesia assistants, and most complaints about peripheral venipuncture were against trainee nurses. The present study findings were significantly different in terms of cannulation site, age, cause of death, type of local complication (p<0.01).

**Conclusion:**

This study shows, as patients become more aware, complaints from physicians about implantation of vascular access, in the judicial authority are also on the rise. Therefore, in choosing these patients, Venice should be treated more carefully.

The US Centers for Disease Control and Prevention estimated 250,000 catheter-related bloodstream infections annually, of whom 20% die from infection persistence (1-3). In a study in Turkey, performed on 74 patients admitted to a medical center- 54% males and 46% females with a mean age of 63 years- six subjects died from cardiac dysrhythmia in the first week and six others in the next 15 days directly from complications of vascular access device implantation (4) . In another study, the thrombosis prevalence was reported 5%-50%, including 35 complications such as infection, sepsis, bleeding, pneumothorax, air embolism, arterial occlusion or nerve blockage, heart perforation, arrhythmia, pleural effusion, edema, and moderate hemorrhage, catheter fracture, catheter occlusion, and thrombophlebitis, so it is important to control complications by clinical and radiological methods after the intervention (5, 6). In a study on late diagnosis and cannulation by inexperienced individuals in an emergency, the incidence of complications was reported higher and more serious (7, 8).

In a meta-analysis of 18 studies with 1646 patients, the application of ultrasound to cannulation led to a significant reduction in complications in both adults and children, although no study was performed on subclavian and femoral catheterization thus far (6, 9). To the best of the authors' knowledge, no studies have been conducted in this regard in forensic commissions, but all previous studies have only been on complications in the medical centers themselves. The purpose of this study was to recognize the complications of implantation of vascular access leading to complaints from patients in the judicial authority.

## Methods

The present descriptive-cross sectional study was performed on all death cases, as well as those complained against medical staff for disability following catheterization, filed with the forensic medical commissions of Tehran, Iran, from 2013 to 2018. Using a researcher-made data collection form, demographic characteristics-i.e., age, patient gender, physician gender, cause of death, underlying disease, catheterization history, type of medical center (educational, non-educational), cannulation agent, type of damage, were extracted from available records. Also, according to the study criteria, all cases without autopsy or incomplete information for any reason or those who died during angiography were excluded from the study. Information extracted from medical files remained confidential, and necessary permissions were obtained from the judicial authority prior to study onset.

Fisher’s exact and Chi-square tests were used to analyze the data in SPSS Version 18. A p-value of <0.01 was considered significant. This article was taken from the research project approved by the Ethics Committee of Shahid Beheshti University of Medical Sciences (ethical code: IR.SBMU.RETECH.REC.1398.475).

## Results

According to the findings, a total of 136 patients were studied; 67% were females. The mean age of the patients was 45±26 years, and the majority was within the age range of 26-70 and were significantly different (p<0.003) (table 1). Totally, 40% of complaints were filed for central venipuncture and 60% for peripheral venipuncture; 90% of the patients had no history of cannulation (p<0.002). All central venipuncture cases were related to the jugular vein and 58% of peripheral venipuncture to the anterior forearm, 29% to cubital fossa (p <0.002), 8% to the wrist, and 5% to legs. In 95% of peripheral venipuncture cases, the initial complication was phlebitis following extravasation and cellulite, and in the remaining 5%, arterial cannulation. Of the total complaints, 48 ​​(35%) were made about death from vascular access, and the rest (n=88, 65%) maim following catheterization. The most significant finding obtained from a patient’s records below 17-year-old was organ failure following peripheral venipuncture in infants (85% of cases below 17). 

**Table 1:** Age Distribution of Complainants Referred to the Forensic Medicine Commission of Tehran Province in 1392-1397(according WHO)

**Age Group, yr**	**Number**	**Percentage**	**P-value**
<17	34	25%	<0.01
18-25	13	9.5%	>0.04
26-65	51	37.5%	<0.003
66-79	38	28%	<0.01
80-90	0	0%	---
Total	136	100	-

**Figure 1:**
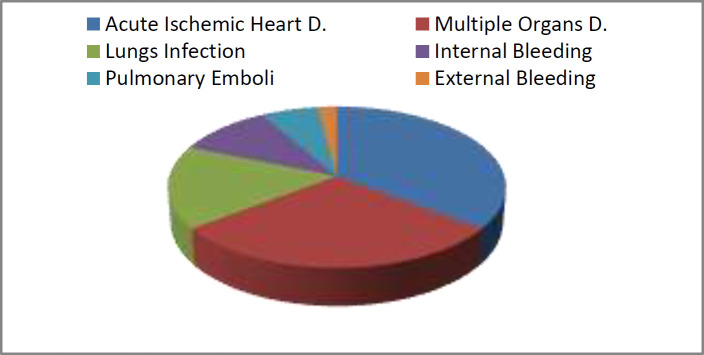
Cause of Death Distribution of Complianants Referred to The Forensic Medicine Commission of Tehran Province

The mean age of dead patients was 56±15 and those with maim 27±20 years. The distribution of physician gender was the same in both groups. In 25% of the cases, CVP line placement was performed by a nurse and in the rest by specialists or their assistants. In 83.5% of death cases, physicians and medical staff were found guilty at exacerbation and acceleration of death-related complications from 3% to 15%, and in 41% of cases filed on maim, clinicians and nurses were found guilty from 2% to 10%. The most common causes of death were acute myocardial infarction (36%), multiple organ failure (29%), pulmonary infection (16%), internal bleeding (11%), and pulmonary embolism (6%), and the least common ones, bleeding from central venous catheter insertion site (2%) (figure 1). I

**Figure 2:**
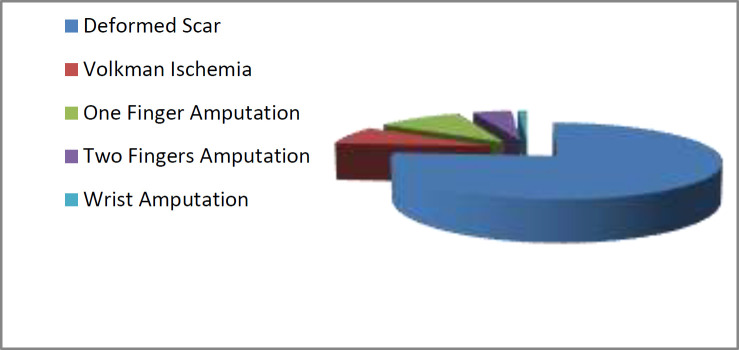
Defect Distribution of Complianats Referred to The Forensic Medicine Commission of Tehran Province in 1392-1397

In maim cases, 76% of complaints were filed about abnormal scars (figure 2). In death cases, 89% of insertions were carried out by general surgery and anesthesia assistants and 11% by nursing staff. In maim cases, 94.5% of complaints were filed against nursing staff about peripheral venipuncture and 4.5% against non-educational physicians.

## Discussion

Unfortunately, no many articles were published academically in this regard in reliable indexes for some reasons, so addressing the complications of vascular access implantation in patients, especially the critically ill ones requiring central vascular access from the jugular vein, subclavian, femoral, or ankle, are somewhat neglected so far by the treatment staff, specifically, the supervisors of medical centers, due to the type of patients and lack of follow-up, but with increasing public awareness due to the expansion of communication, complaints about such surgical procedures are increasing in Iran and the world(10, 11). In a study in Turkey at a medical center on 74 patients, six died from catheterization complications (4); however, the present study was more comprehensive, and 15 cases died directly from catheterization complications (bleeding), while no direct cause was mentioned in the study from Turkey. In a study in Isfahan, Iran (2011), on 96 patients hospitalized in the burn unit, similar to the present study, the majority were males aged 20-50 years. Out of 26 cases with implantation complications, most were related to insertion failure (15.6%), hematoma (6.3%), cannulation (4.2%), and pneumothorax (1.04%)(12); however, in the present study, including all cases, especially infants from surgery / internal medicine/ pediatric fields, death cases were also analyzed. In another study, local complications were more common in patients with diabetes and obesity, as well as smokers, so that 5.7% of them required surgery due to exacerbation of local complications. In this study, similar to the present research, most patients were males with an underlying disease; however, inconsistent with the present study, the mortality rate was also reported. In addition, the access sites in peripheral venipuncture were mostly the anterior forearm and cubital fossa. The most common primary complication was phlebitis and cellulite in that study, consistent with the present study results. No further follow-up was carried out regarding the prognosis of these primary complications. One of the present study’s weaknesses, compared to the latter study mentioned, was the small sample size and inability to evaluate cases with cannulation failure (301 vs. 136 patients) (13). Although all patients in the present study had underlying diseases, inconsistent with the mentioned study, the type of permanent and definite complications, such as amputation and formation of scars requiring reconstructive surgery, was defined.

 Similar to other studies, with increasing age, the rate of local complications increased in the present study; in other words, the mortality rate was consistent with patients’ age increase. To the best of the authors' knowledge, no study thus far examined complaints filed on complications of vascular access devices implantation with forensic medicine authorities, and they only reviewed patients' medical records (2, 3, 14). In conclusion According to the present study’s findings, the need for timely and accurate documentation of events and their reflection in the patient file in case of complications during venipuncture was quite evident to explore the complaint in cases where the treatment staff did not take necessary measures to resolve their suspicions in incidence or exacerbation of complications, leading to death following implantation of central venous access devices, resulting in conviction in the medical commission.

**Recommendation**: 

1) - To follow up and prevent the loss of the rights of the treatment staff, the patient, or the deceased, it is reminded that the medical staff document in writing all the unwanted events that may occur to them and also timely inform the authorities to solve the problem.

2) - After performing the venipuncture, be ensure of the correct placement of the catheter in the vein, and in case of doubt, take the necessary measures, including consultation with other qualified personnel.

3) - The need to purchase or extend professional liability insurance, even those who are confident in their skills and profession; bear in mind that the occurrence of complications is unpredictable in any medical or nursing measures.

## References

[1] WHO ( 2010). WHO guidelines on drawing blood: best practices in phlebotomy.

[2] Webster J, Osborne S, Rickard CM, Marsh N (2019). Clinically‐indicated replacement versus routine replacement of peripheral venous catheters. Cochrane Database Syst Rev.

[3] Mohan P, Kumar R (2019). Strengthening primary care in rural India: Lessons from Indian and global evidence and experience. J Family Med Prim Care.

[4] Barik D, Thorat A (2015). Issues of unequal access to public health in India. Front Public Health.

[5] Galena HJ (1992). Complications occurring from diagnostic venipuncture. J Fam Pract.

[6] Xu X, Wang B, Ren C (2017). Age-related impairment of vascular structure and functions. Aging Dis.

[7] Dychter SS, Gold DA, Carson D, Haller M (2012). Intravenous therapy: a review of complications and economic considerations of peripheral access. J Infus Nurs.

[8] Prakash S, Arora G, Rani HS (2015). Peripheral venous access in the obese patient. Indian J Anesth.

[9] Fields JM, Piela NE, Au AK, Ku BS (2014). Risk factors associated with difficult venous access in adult ED patients. Am J Emerg Med.

[10] Kagel EM, Rayan GM (2004). Intravenous catheter complications in the hand and forearm. J Trauma.

[11] O'Grady NP, Alexander M, Burns LA (2011). Guidelines for the prevention of intravascular catheter-related infections. Clin Infect Dis.

[12] Sayyadi M, Yosefzadeh M, Babakhani A, Beigi A (2011). Evaluating the central venous catheter complications of burned patients in Imam Mousa Kazem Hospital of Isfahan during 2007-8. Feyz, J Kashan Univ Med Sci.

[13] Chaudhary MK, Dhakaita S, Ray R, Baruah TD (2020). Local complications of intravenous access–an often underestimated entity. J Family Med Prim Care.

[14] Schmid MW (2000). Risks and complications of peripherally and centrally inserted intravenous catheters. Crit Care Nurs Clin North Am.

